# Utilization of machine learning models in predicting caries risk groups and oral health-related risk factors in adults

**DOI:** 10.1186/s12903-024-04210-z

**Published:** 2024-04-08

**Authors:** Burak Tunahan Çiftçi, Firdevs Aşantoğrol

**Affiliations:** Department of Oral and Maxillofacial Radiology, Faculty of Dentistry, Gaziantep University, Gaziantep, Türkiye 27310

**Keywords:** Oral health, Dental caries susceptibility, Machine learning algorithms, Prediction

## Abstract

**Background:**

The aim of this study was to analyse the risk factors that affect oral health in adults and to evaluate the success of different machine learning algorithms in predicting these risk factors.

**Methods:**

This study included 2000 patients aged 18 years and older who were admitted to the Department of Oral and Maxillofacial Radiology, Faculty of Dentistry, Gaziantep University, between September and December 2023. In this study, patients completed a 30-item questionnaire designed to assess the factors that affect the decayed, missing, and filled teeth (DMFT). Clinical and radiological examinations were performed, and DMFT scores were calculated after completion of the questionnaire. The obtained data were randomly divided into a 75% training group and a 25% test group. The preprocessed dataset was analysed using various machine learning algorithms, including naive Bayes, logistic regression, support vector machine, decision tree, random forest and Multilayer Perceptron algorithms. Pearson's correlation test was also conducted to assess the correlation between participants' DMFT scores and oral health risk factors. The performance of each algorithm was evaluated to determine the most appropriate algorithm, and model performance was assessed using accuracy, precision, recall and F1 score on the test dataset.

**Results:**

A statistically significant difference was found between various factors and DMFT-based risk groups (*p* < 0.05), including age, sex, body mass index, tooth brushing frequency, socioeconomic status, employment status, education level, marital status, hypertension, diabetes status, renal disease status, consumption of sugary snacks, dry mouth status and screen time. When considering machine learning algorithms for risk group assessments, the Multilayer Perceptron model demonstrated the highest level of success, achieving an accuracy of 95.8%, an F1-score of 96%, and precision and recall rates of 96%.

**Conclusions:**

Caries risk assessment using a simple questionnaire can identify individuals at risk of dental caries, determine the key risk factors, provide information to help reduce the risk of dental caries over time and ensure follow-up. In addition, it is extremely important to apply effective preventive treatments and to prevent the general health problems that are caused by the deterioration of oral health. The results of this study show the potential of machine learning algorithms for predicting caries risk groups, and these algorithms are promising for future studies.

## Background

Oral health is a critical aspect of general health that affects many daily activities, including eating, speaking, social relationships and appearance [1]. The World Health Organization has identified oral health as one of the top public health priorities worldwide due to its significant impact on people's daily lives [2]. Between 60 and 90% of children in the world and almost all adults suffer from dental caries. Furthermore, approximately 30% of individuals aged 65–74 have no natural teeth [3].

Dental caries is the result of bacterial fermentation of dietary carbohydrates, which produces acidic by-products that cause localized destruction of dental hard tissues [4]. The hard dental tissues show signs of demineralization, but the disease process begins within the bacterial biofilm covering the tooth surface. Dental caries is a multifactorial disease that originates from microbiological changes within the complex biofilm. It is influenced by various factors, such as saliva flow and composition, fluoride exposure, dietary sugar consumption, and preventive behaviours. The disease is reversible in its early stages and can be controlled at any stage, even if part of the dentin or enamel is damaged, providing that the biofilm can be adequately eliminated. Dental caries is a chronic disease that is typically slow in progression for the majority of people. Both the crowns and roots of deciduous and permanent teeth can be affected by dental caries [5].

Various factors, such as age and sex [6], oral hygiene [7], overweight status [8], hypertension [9], diabetes status [10], consumption of alcohol [11], smoking status [12], type of diet [13], amount and content of saliva [14], history of chemotherapy [15] and radiotherapy [16], malocclusion [17], educational level [18], and socioeconomic status [19], affect the incidence of dental caries.

The decayed, missing and filled teeth (DMFT) index is a commonly used and straightforward tool in epidemiological studies of dental caries. The DMFT index assesses dental health status based on the number of decayed, missing, and filled teeth [20]. This index is employed to evaluate and monitor oral health interventions in the community through the development of policies and programmes in this area [21, 22].

Artificial intelligence refers to the capability of computers to learn by inputting data. Its objective is to identify an optimal and adaptable approach to problem solving without human intervention [23]. Machine learning, one of the sub-branches of artificial intelligence, utilizes methods of computation and data training. It analyses the input information and processes the information obtained from accumulated experience. Gathering of experience, or active learning, is the basis of machine learning. In practice, this is how the computers improve their performance by learning from the input data and building a specific model [24].

The use of machine learning has recently been extended to different clinical specialities in dentistry. A wide range of tools are available to support diagnosis and prognosis and improve clinical decisions [25]. In the literature, there are different studies [26–34] in which machine learning models are used for the prediction of risk factors; however, all of those studies were conducted in paediatric patients, except for the study by Hung et al. [35] on root caries prediction. The aim of this study was to analyse the risk factors thought to be effective for oral health in adults and to evaluate the success of various machine learning algorithms in predicting the risk factors associated with patients’ oral health.

## Methods

### Patient selection

The study included 2000 patients aged 18 years and older who were admitted to the Department of Oral and Maxillofacial Radiology, Faculty of Dentistry, Gaziantep University, between September and December 2023. This study was reviewed by the Gaziantep University Clinical Studies Ethics Committee and approved with decision number 2023/311. The study was conducted following the principles of the Declaration of Helsinki.

### Data collection

The DMFT index is the main indicator of caries experience in the community. It has been used for more than half a century. The DMFT (decayed, missing, filled teeth) index was recommended by the WHO and first described by Klein and Palmer in 1938 [36]. This index is used to assess the amount of decayed, missing and filled teeth in an individual. 28 permanent teeth are quantified and third molars are usually not included. An individual's DMFT score ranges from 0 to 28. A score of 0 means that no teeth are decayed, missing, or filled. A score of 28 means that all teeth are affected. A tooth is quantified as decayed if it is both restored and decayed.

In this study, patients completed a 30-item questionnaire designed to assess the factors that affect the DMFT. The following inquiries were made in the questionnaire: age, sex, body mass index, frequency of tooth brushing, socioeconomic status, employment status, education level, marital status, alcohol and smoking status, hypertension, diabetes status, chronic obstructive pulmonary disease (COPD), heart and renal diseases, stroke status, sugary snack consumption and frequency, dental flossing, malocclusion, history of chemotherapy and radiotherapy, dry mouth, visual impairment, attention deficit hyperactivity disorder (ADHD), memory impairment, number of dental visits in the last year, time spent in front of television, telephone and computer, difficulty in performing daily activities, and walking impairment. Clinical and radiological examinations were performed, and DMFT scores were calculated after completion of the questionnaire. The DMFT calculation excluded third molars, congenitally missing teeth, supernumerary teeth, and teeth extracted for reasons other than decay, such as trauma or orthodontic purposes, as well as teeth with fillings for non-decay reasons, such as aesthetic purposes. The number of filled, decayed, and extracted teeth were calculated separately and then summed. To minimize the possibility of errors and bias in DMFT calculations, all examinations were carried out by the single observer, who was an experienced oral and maxillofacial radiologist. The examinations were conducted in good lighting conditions using a flat mirror and an examination probe to ensure reliable and consistent results. Patients were classified according to the DMFT score as follows.

- Low risk: DMFT score < 4
- Moderate risk: 4 ≤ DMFT score ≤ 8
- High risk: Patients with a DMFT score > 8 [37].

### Data preprocessing

In the study, the input data used to train the model were encoded into numerical codes according to certain criteria. This coding scheme is as follows: 1 for no and 2 for yes in yes and no questions; 1 for male and 2 for female in gender question; 1 for not working, 2 for working and 3 for retired in determining employment status; 1 for single and 2 for married in determining marital status. For the other level questions, 1 for the lowest and 3 for the highest score for 3-point classifications; 1 for the lowest and 2 for the highest score for 2-point classifications. The DMFT risk groups, which are the output of our study, were coded as 1 for low risk, 2 for moderate risk and 3 for high risk.

Independent variables were standardised with a mean value of 0 and a standard deviation of 1 in order to prevent large effects due to scale differences, to prevent model bias and to achieve generalisable results.

### Model development

**Fig. 1:**
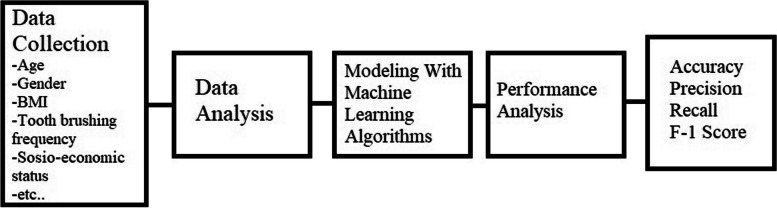
Flow diagram of the study for predicting caries risk groups and oral health-related risk factors using five different machine learning algorithms

The obtained data were randomly divided into a 75% training group and a 25% test group. The open-source version (v3.11) of the Python programming language and the IPython library were used for the model development process. Model training was carried out on a computer equipped with an NVIDIA GeForce MX 330 graphics card with 8 GB of RAM. The preprocessed dataset was analysed using a range of machine learning algorithms, including naive Bayes (NB), logistic regression (LR), support vector machine (SVM), decision tree (DT), random forest (RF) and Multilayer Perceptron (MLP) algorithms. The study methodology is summarized and presented as a template in Fig. 1.

#### Naive bayes

The NB classifier is a simple and easy-to-use probabilistic classifier that relies on Bayes' theorem. It assumes that each attribute variable is independent. This classifier can be successfully trained using supervised learning and can also be applied to complicated real-world situations. The main advantage of NB is that it requires only a small amount of training data, which is essential for characterization and classification [38]. The classification is performed using the Bayesian principle to calculate the probability of the class name C, considering the particular instance X_1_…X_n_, by the following formula: ${\text{P}}({\text{C}}={\text{c}}|{\text{Xl}}={\text{xl}},\dots ,\mathrm{ Xn}={\text{xn}})$. The classifier can be defined as Classify (A_1_…. A_n_) = argmax P(C = c)∏^n^_i=1_*p*(A_i_ = ai|C = c), where A1…An = attribute variables and C = class name [39].

#### Logistic regression

LR is a widely used model in binary classification problems, where the dependent variable takes on only two values (0 and 1). It is also commonly applied in various other fields, including machine learning [40]. LR is a prediction analysis which explains the relationships between a binary dependent variable and a series of independent variables. For equation a + bx, the probability of an event occurring is as follows.

$${\text{p}}={\text{e}}\frac{a+bx }{1+e}a+bx$$

The logit function with the probability of nonoccurrence of the event being 1-p is as follows.

$${\text{logit}}({\text{p}})=\mathrm{ln }(\frac{{\text{p}}}{1-{\text{p}}})$$

LR produces the coefficients of a formula for estimating the logit transformation.

### Support vector machine

The SVMs were developed by Alexey Ya. Chervonenkis and Vladimir N. Vapnik back in 1963 [41]. Since the inception of SVMs, this methodology has been widely adopted for addressing problems related to image, hypertext, and text segregation and categorization. These models represent a high level of sophistication and find utility in handwritten text recognition as well as protein sorting in biological laboratories. They have been effectively employed in diverse domains, such as autonomous vehicles, conversational agents (chatbots), and facial recognition [42]. As one of the most prevalent supervised learning algorithms, the SVM algorithm is designed to handle regression and classification tasks. The main purpose of SVMs is to define an optimal decision boundary, termed a hyperplane, which divides an n-dimensional space effectively into separate classes, facilitating the accurate categorization of data points. In the SVM algorithm, critical vector points known as support vectors are identified and play a crucial role in the definition of the appropriate hyperplane. SVM applications encompass a wide array of tasks, including facial detection, image classification, and text categorization [43].

#### Desicion tree

**Fig. 2:**
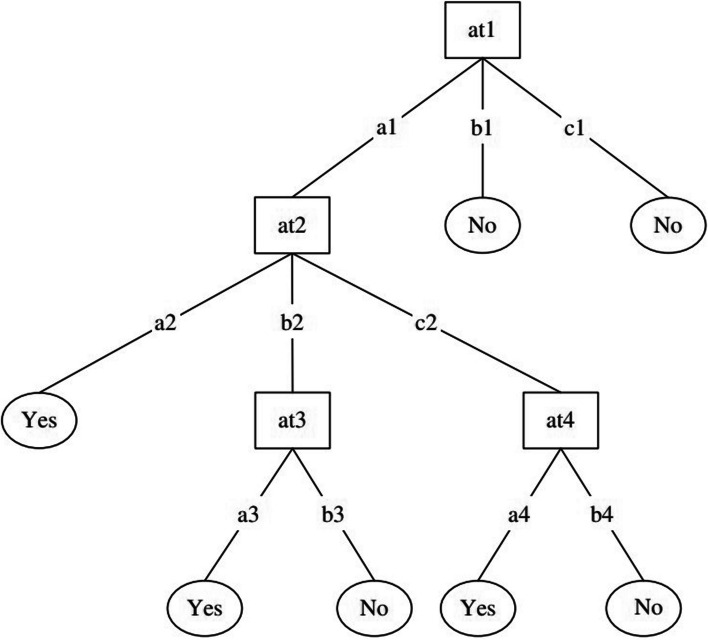
An example of a decision tree for the training set from Table 1 [45]

A DT functions as a classifier by recursively dividing the instance space. A DT comprises nodes that construct a rooted tree, which means that it is a directed tree featuring a node termed the "root" without any incoming edges (Fig. 2). There is exactly one incoming edge on all the other nodes. A node with outwards edges is referred to as an internal or testing node. Conversely, all other nodes are denoted as leaves, alternatively recognized as terminal or decision nodes. A DT's internal nodes divide the instance space into subspaces based on a specific function that is discrete with respect to the values of the input attributes. In the most straightforward and commonly occurring scenario, each test examines a single attribute, thereby partitioning the instance space according to the value of the attribute. The condition applies to a range if the attribute is numeric. Each leaf is associated with one class that signifies the most suitable target value. Otherwise, the leaf may contain a probability vector signifying the likelihood of the target attribute to have a particular value. The instances are categorized by guiding them from the root of the tree down to a leaf, depending on the results of the testing along the route [44].

Figure 2 [45] shows an example of a DT for the training set of Table 1.

**Table 1:** Training set for the decision tree machine learning model

Models	Parameters	Optimal Values
Naive Bayes	var_smoothing	1e-09
Logistic Regression	C	0.01
	penalty	l2
Support Vector Machine	C	1
	Kernel	1
	Gamma	rbf
Decision Tree	criterion	Gini
	max_depth	None
	min_samples_leaf	1
	min_samples_split	2
Random Forest	criterion	entropy
	max_depth	None
	min_samples_leaf	1
	min_samples_split	2
	n_estimators	15
Multilayer Perceptron	alpha	0.001
	hidden_layer_sizes	100,
	learning_rate	constant
	max_iter	1000

#### Random forest

**Fig. 3:**
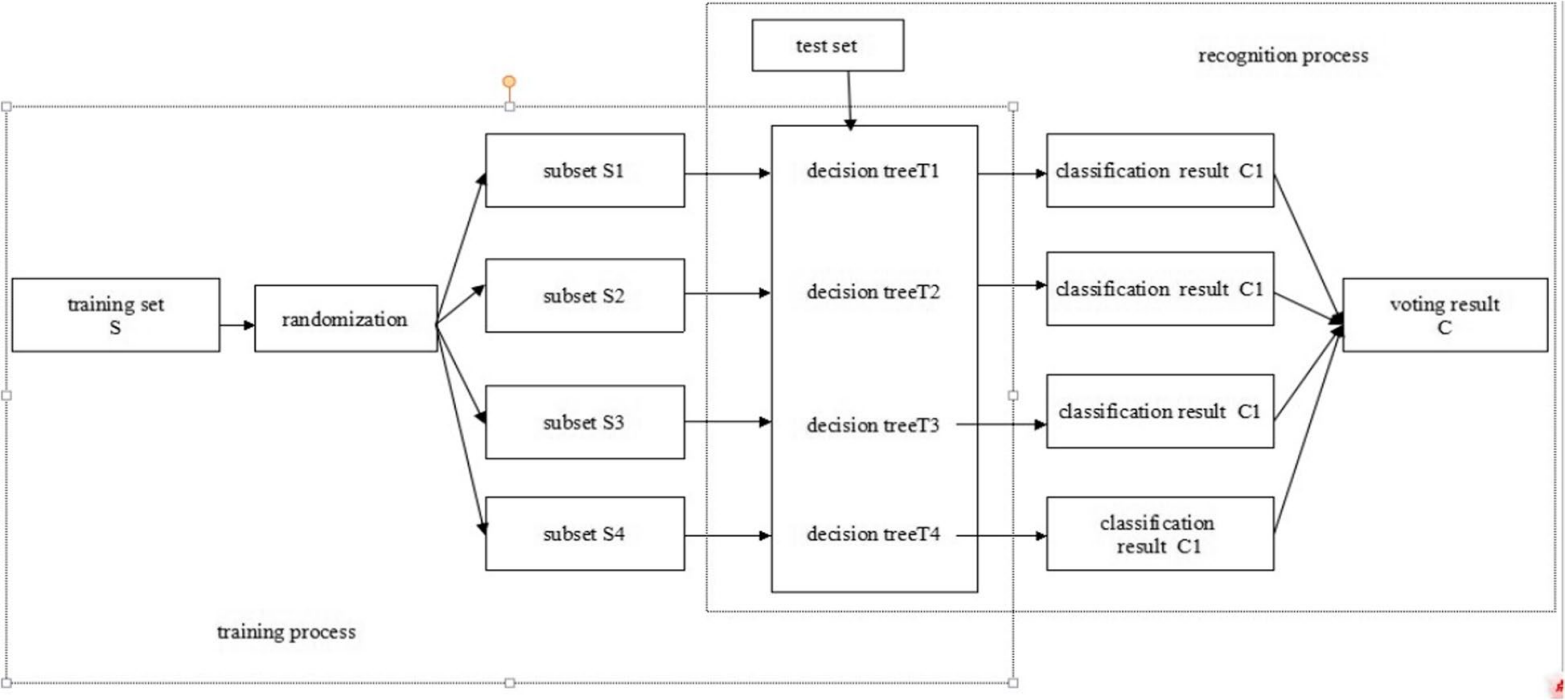
The structure of the random forest classifier [47]

The RF was introduced by Leo Breiman in 2001 [46]. It consists of a number of basic classifiers (decision trees) that are independent of one another. To classify a test sample, the RF algorithm aggregates the results of each individual classification, and the class label of the sample is determined by a majority vote. Figure 3 [47] illustrates the whole process of classification using the RF algorithm. A large number of decision trees are used in the RF algorithm. The construction process introduces a random operation, which includes the selection of a subset of samples and features, to ensure the independence of each decision tree, improving the accuracy of classification and achieving a more generalized ability [46].

#### Muti-layer perceptron

**Fig. 4:**
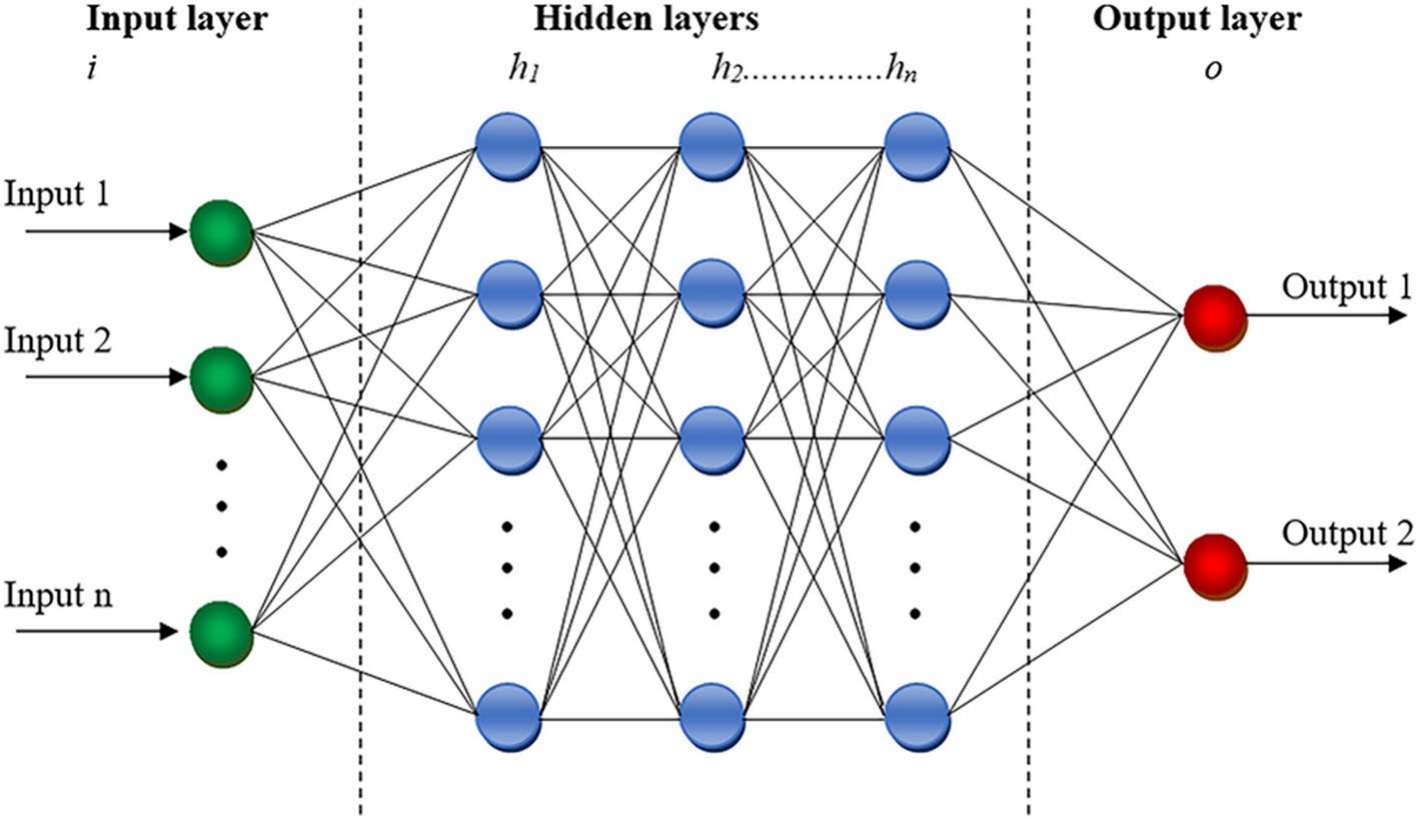
Architecture of multilayer perceptron artificial neural network (MLP-ANN) [48]

The MLP network represents a type of feedforward artificial neural network (ANN) characterized by three primary layers: an input layer, one or more hidden layers, and an output layer (Fig. 4) [48]. The hidden layer consists of neurons with activation functions defining their behavior. Inputs from the input layer pass through the initial hidden layer, where the number of nodes aligns with the input features [49]. In this layer, the weighted sum of inputs, adjusted by bias values, is calculated using a specified equation [50].

$$\mathrm{V }=\mathrm{ X}1 *\mathrm{ W}1 +\mathrm{ X}2 *\mathrm{ W}2 + . . . +\mathrm{ Xn }*\mathrm{ W n }+\mathrm{ Bias}.$$

Within each hidden layer node, an activation function such as Sigmoid or ReLU is applied to determine the node's output, which is then forwarded to the subsequent layer. The output layer, equipped with an activation function tailored to the desired output type, produces the final result through a process known as forward feeding [47]. The obtained output is evaluated by calculating the error rate, representing the difference between the expected target and the actual output. Minimizing this error rate is crucial.

To refine the MLP's performance, backpropagation is employed during each epoch, adjusting the network weights based on the previously computed error rate [50]. MLP networks are specifically designed to address non-linearly separable problems. Notably, they find widespread application in pattern recognition and play a significant role in predicting and diagnosing diseases [51].

### Validation and hyperparameter tuning of models

In the study, the fivefold cross-validation method was used to evaluate the accuracy of the model and to prevent overfitting. This method divides the dataset into five different subsets and tests the generalisation ability of the model by training and evaluating each subset separately. The Grid Search method was used for hyperparameter optimisation. This method determines the parameter set that provides the best performance by trying different combinations of parameters that affect the model performance. This allowed us to optimise our model and obtain the best results. Table 2 shows the hyperparameter values selected as a result of Grid Search for each algorithm.

**Table 2:** Optimizable parameters for different models

at1	at2	at3	at4	Class
a1	a2	a3	a4	Yes
a1	a2	a3	b4	Yes
a1	b2	a3	a4	Yes
a1	b2	b3	b4	No
a1	c2	a3	a4	Yes
a1	c2	a3	b4	No
b1	b2	b3	b4	No
c1	b2	b3	b4	No

### Feature selection

The feature importance function in Python's sklearn library was used to obtain and graph the most significant features for determining the DMFT classification model.

### Statistical analysis

**Fig. 5:**
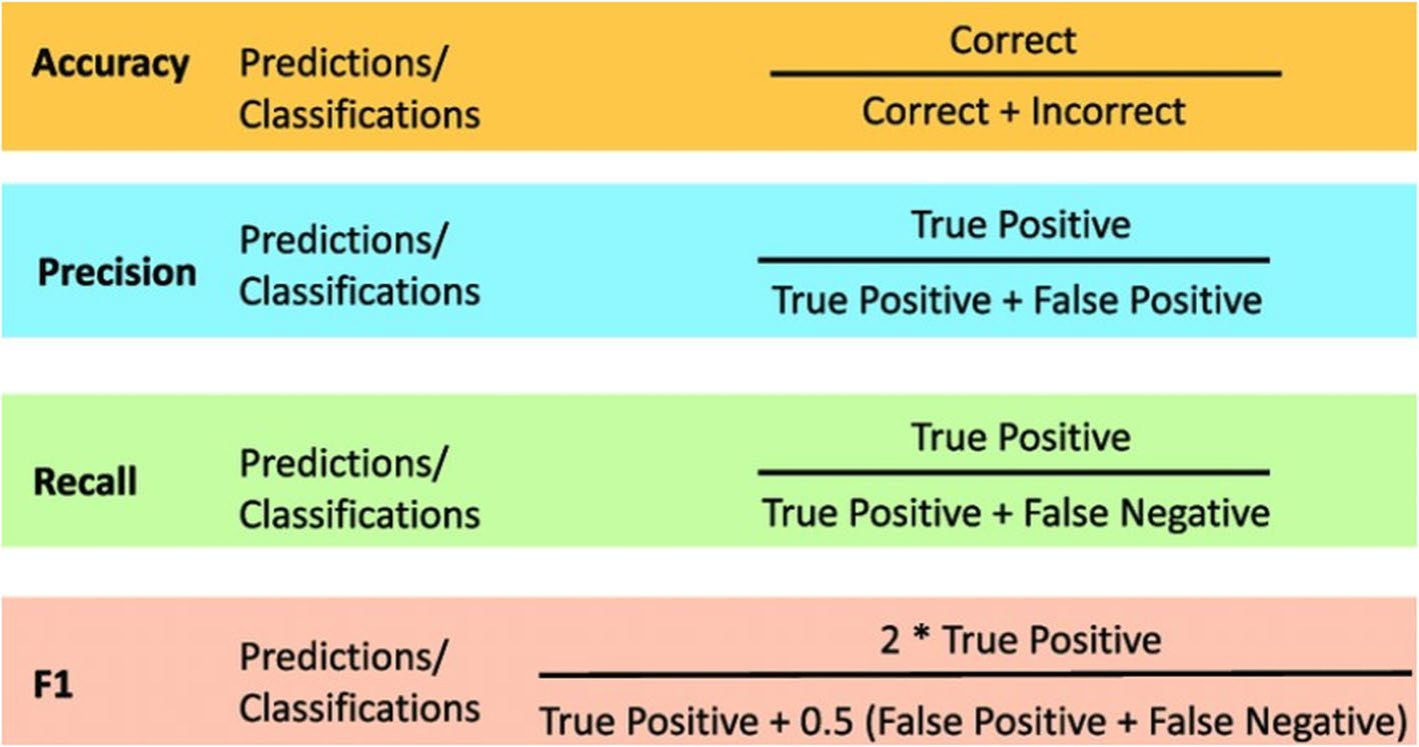
Evaluation metrics for the classifications [52]

To investigate the correlation between the DMFT score and oral health risk factors, Pearson's correlation test was performed. The performance of each algorithm was evaluated to determine the most appropriate algorithm, and the performance of the models was analysed using accuracy, precision, recall and F1 score on the test dataset. The statistical significance level was determined as *p* < 0.05. The formulae of these evaluation metrics are shown in Fig. 5 [52].

## Results

Of the 2000 patients who participated in our study, 546 (27.3%) were in the low-risk group, 850 (42.5%) were in the moderate-risk group and 604 (30.2%) were in the high-risk group. Of these patients, 858 (42.9%) were male and 1142 (57.1%) were female. A total of 890 patients (44.5%) were aged between 18 and 30 years, 858 patients (42.9%) were aged between 30 and 50 years, and 252 patients (12.6%) were aged older than 50 years.

**Fig. 6:**
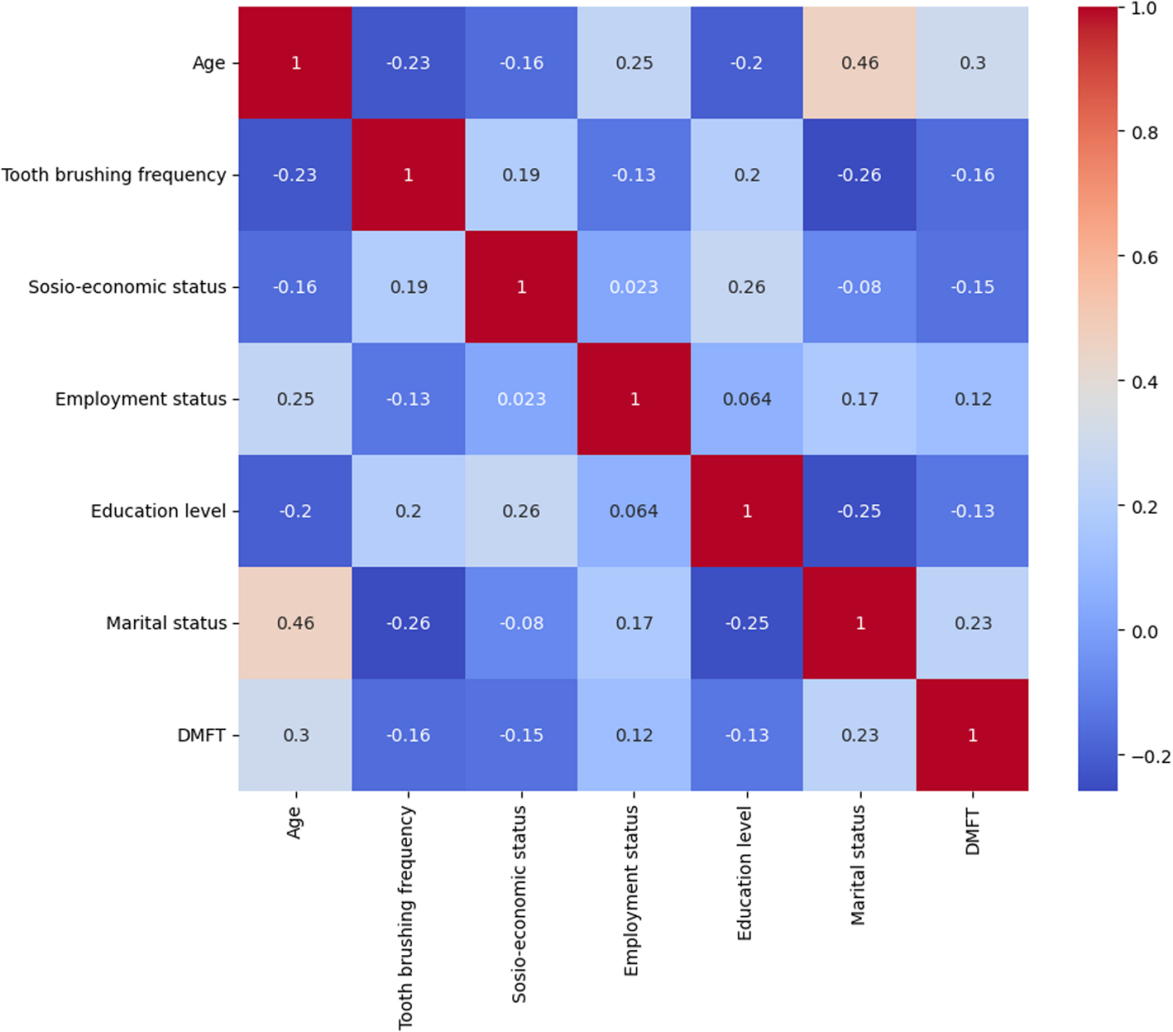
Correlation heatmap showing the relationships between oral health-related risk factors

The distributions of oral health risk groups according to DMFT score and their statistical significance levels are shown in Table 3. According to these data, significant differences were found in age; sex; body mass index; tooth brushing frequency; socioeconomic status; employment status; education level; marital status; hypertension; diabetes; renal disease; consumption of sugary snacks; dry mouth; and time spent in front of television, telephone and computer and in the DMFT risk group (*p* < 0.05). A correlation heat graph showing the relationships between these values is shown in Fig. 6. There was a low negative correlation between age and the consumption of sugary snacks (*r* = -0.24) and a low negative correlation between tooth brushing frequency and marital status (*r* = -0.25), while there was a low positive correlation between tooth brushing frequency and education level (*r* = 0.20). There was a moderate positive correlation between age and marital status (*r* = 0.46).

**Table 3:** The distribution of individuals' oral health-related risk factors within the caries risk groups based on DMFT scores

Variables		Low Risk(DMFT < 4)		Moderate Risk(4 ≤ DMFT ≤ 8)		High Risk (DMFT > 8)		*p* Value
		*n* = 546		*n* = 850		*n* = 604		
		n	%	n	%	n	%	
Age	< 30	342	62.6	390	45.8	158	26.1	***p* < 0.00001**
	30–50	168	30.7	386	45.4	304	50.3	
	> 50	36	6.5	74	8.7	142	23.5	
Gender	Men	208	38	360	42.3	290	48	***p* = 0.01**
	Women	338	61.9	490	57.6	314	51.9	
BMI	< 30 kg/m^2^	422	77.2	656	77.1	422	69.8	***p* = 0.03**
	≥ 30 kg/m^2^	124	22.7	194	22.8	182	30.1	
Tooth brushing frequency	≤ 1	272	50.9	488	57.4	430	71.1	***p* < 0.00001**
	2	238	43.5	312	36.7	166	27.4	
	≥ 3	30	5.4	50	5.8	8	1.3	
Socioeconomic status	Low	76	13.9	182	21.4	186	30.7	***p* < 0.00001**
	Moderate	454	83.1	650	76.4	404	66.8	
	High	16	2.9	18	2.1	14	2.3	
Employment status	Not working	352	64.4	522	61.4	320	52.9	***p* = 0.0001**
	Employed	178	32.6	308	36.2	226	37.4	
	Retired	16	2.9	20	2.3	58	9.6	
Education level	≤ Middle school	252	46.1	398	46.8	394	65.2	***p* = 0.00002**
	High school	202	36.9	292	34.3	146	24.3	
	≥ College	92	16.8	160	18.8	64	10.5	
Marital status	Single	316	57.8	380	44.7	166	27.4	***p* < 0.00001**
	Married	230	42.1	470	55.2	438	72.5	
Alcohol use	Yes	38	6.9	84	9.8	32	5.2	*p* = 0.41
	No	508	93	766	90.1	572	94.7	
Tobacco use	Yes	118	21.6	266	31.2	154	25.4	*p* = 0.33
	No	428	78.3	584	68.7	450	74.5	
Hypertension	Yes	34	6.2	50	5.8	64	10.5	***p* = 0.04**
	No	512	93.7	800	94.1	540	89.4	
Diabet	Yes	22	4	34	4	58	9.6	***p* = 0.003**
	No	524	95.9	816	96	546	90.3	
COPD	Yes	6	1	6	0.7	4	0.6	*p* = 0.56
	No	540	98.9	844	99.2	600	99.3	
Heart failure	Yes	16	2.9	14	1.6	30	4.9	*p* = 0.13
	No	530	97	836	98.3	574	95	
Stroke	Yes	10	1.8	18	2.1	6	0.9	*p* = 0.42
	No	536	98.1	832	97.8	598	99	
Renal failure	Yes	6	1	32	3.7	28	4.6	***p* = 0.01**
	No	540	98.9	818	96.2	576	95.3	
Consumption of sugary snacks	Yes	362	66.3	542	63.7	332	54.9	***p* = 0.04**
	No	184	33.6	308	36.2	272	45	
Frequency of snack consumption	< 2 per day	328	60	510	60	410	67.8	*p* = 0.05
	≥ 2 per day	218	39.9	340	40	194	32.1	
Use of dental floss	Yes	70	12.8	104	12.2	64	10.5	*p* = 0.40
	No	476	87.1	746	87.7	540	89.4	
Malocclusion	Yes	90	16.4	156	18.3	72	11.9	*p* = 0.12
	No	456	83.5	694	81.6	532	88	
Chemotherapy history	Yes	2	0.3	16	1.8	8	1.3	*p* = 0.33
	No	544	99.6	834	98.1	596	98.6	
Radiotherapy history	Yes	8	1.4	18	2.1	24	3.9	*p* = 0.05
	No	538	98.5	832	97.8	580	96	
Dry mouth	Yes	132	24.1	210	24.7	208	34.4	***p* = 0.005**
	No	414	75.8	640	75.2	396	65.5	
Visual impairment	Yes	94	17.2	140	16.4	134	22.1	*p* = 0.11
	No	452	82.7	710	83.5	470	77.8	
ADHD	Yes	32	5.8	40	4.7	36	5.9	*p* = 0.93
	No	514	94.1	810	95.2	568	94	
Memory impairment	Yes	36	6.5	54	6.3	38	6.2	*p* = 0.88
	No	510	93.4	796	93.6	566	93.7	
Dentist visit in the last 1 year	0	310	56.7	442	52	334	55.2	*p* = 0.75
	≥ 1	236	43.2	408	48	270	44.7	
Time spent in front of the TV, phone or computer	≤ 3 h	184	33.6	326	38.3	278	46	***p* = 0.003**
	> 3 h	362	66.3	524	61.6	326	53.9	
Experiencing difficulties in performing daily life activities	Yes	80	14.6	144	16.9	114	18.8	*p* = 0.17
	No	466	85.3	706	83	490	81.1	
Walking disorder	Yes	38	6.9	62	7.2	68	11.2	*p* = 0.05
	No	508	93	788	92.7	536	88.7	

*DMFT *Decayed missing and filled teeth, *BMI* Body mass index, *kg/m*^*2*^ Kilogram/meter square, *COPD* Chronic obstructive pulmonary disease, *ADHD* Attention deficit hyperactivity disorder

**Fig. 7:**
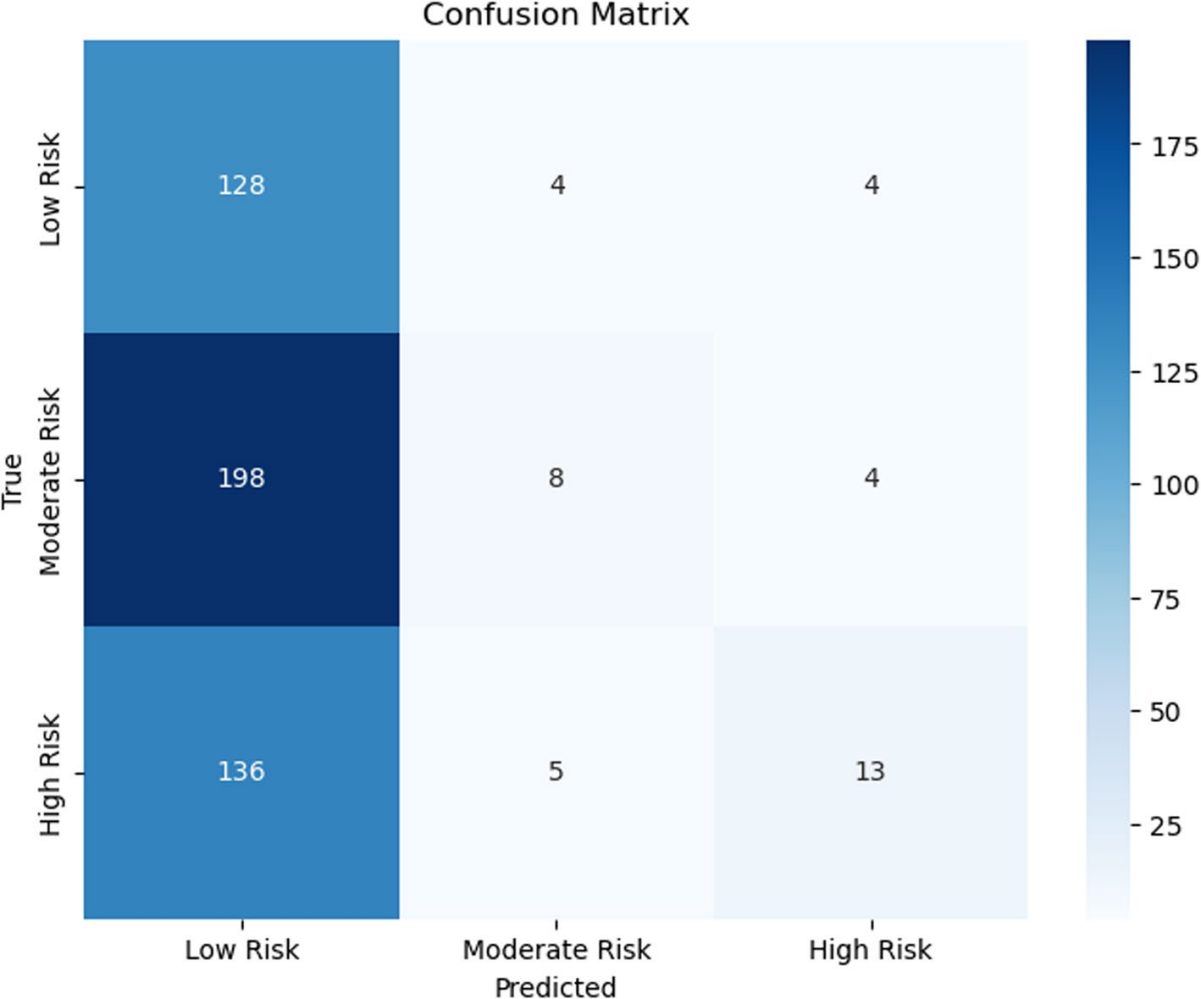
Confusion matrix plot for naive bayes machine learning model for predicting risk groups based on DMFT

**Fig. 8:**
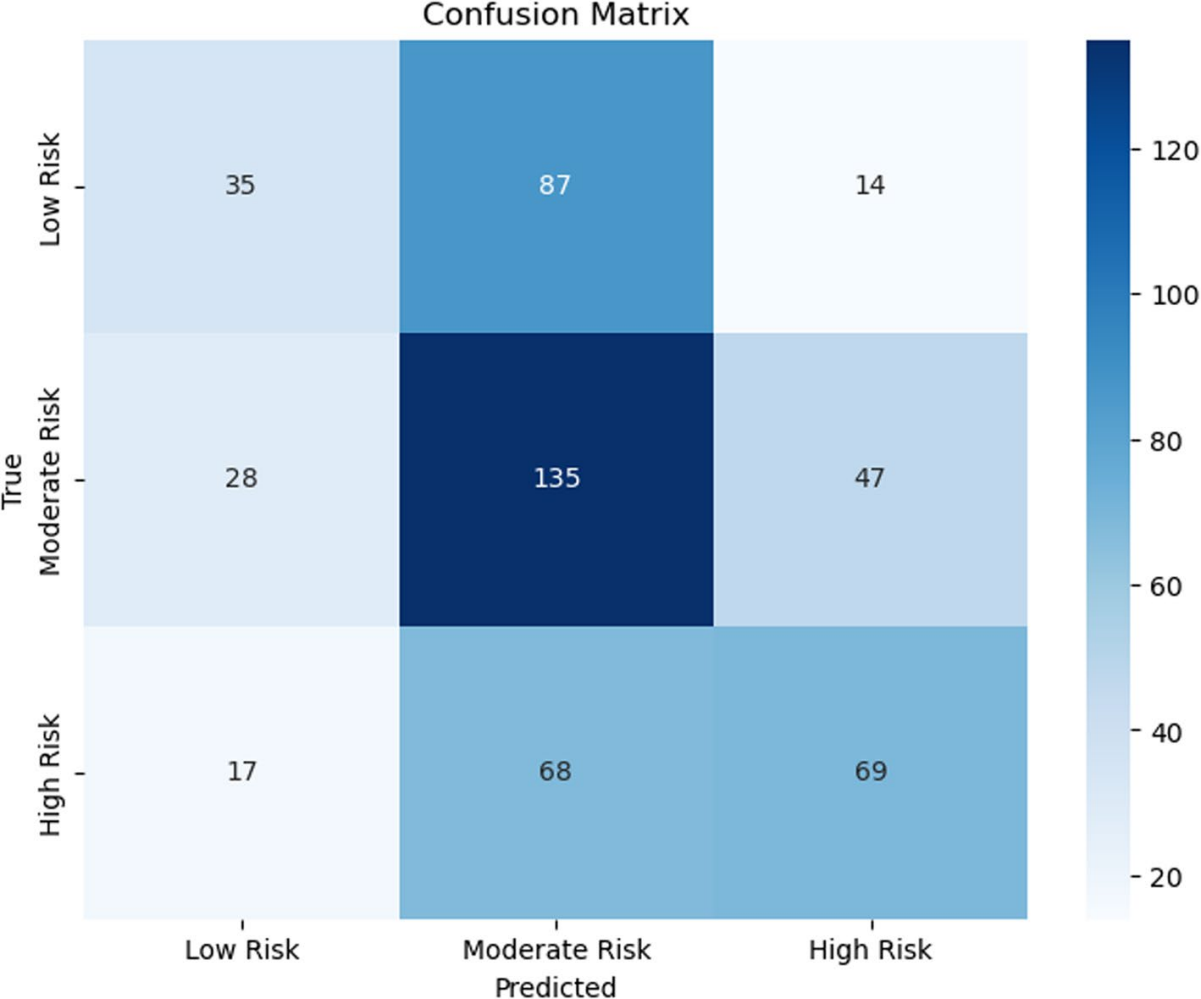
Confusion matrix plot for the logistic regression machine learning model for predicting risk groups based on DMFT

**Fig. 9:**
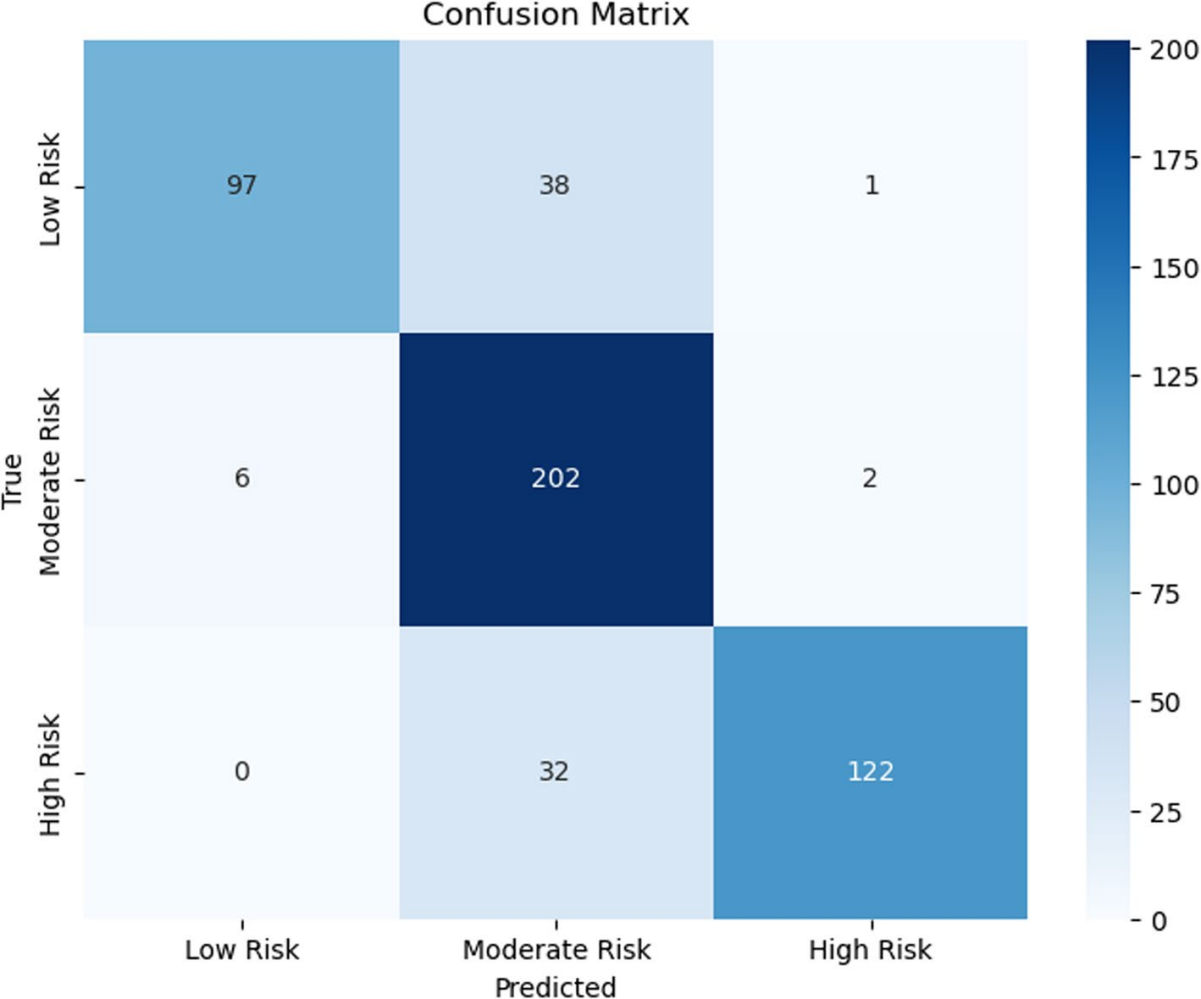
Confusion matrix plot for the support vector machine learning model for predicting risk groups based on DMFT

**Fig. 10:**
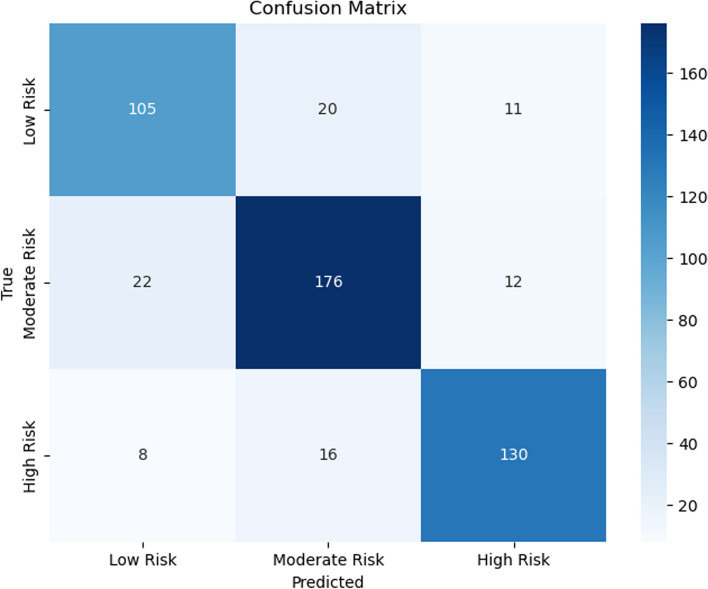
Confusion matrix plot for the decision tree machine learning model for predicting risk groups based on DMFT

**Fig. 11:**
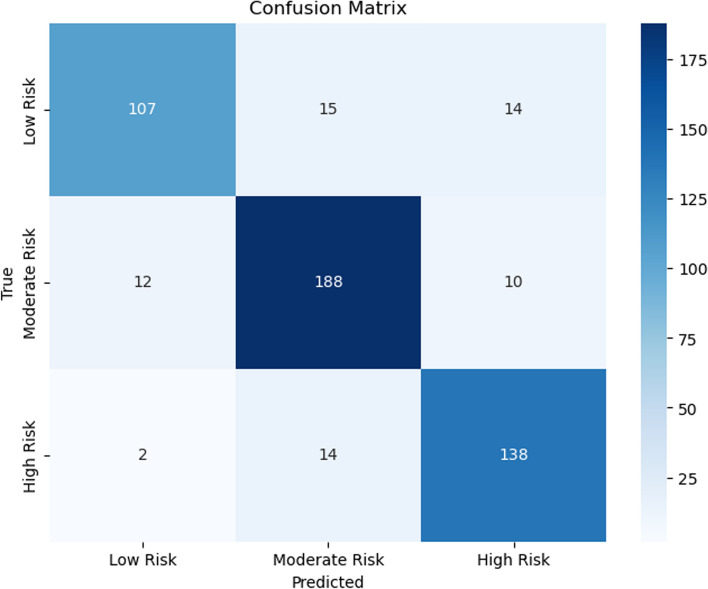
Confusion matrix plot for the random forest machine learning model for predicting risk groups based on DMFT

**Fig. 12:**
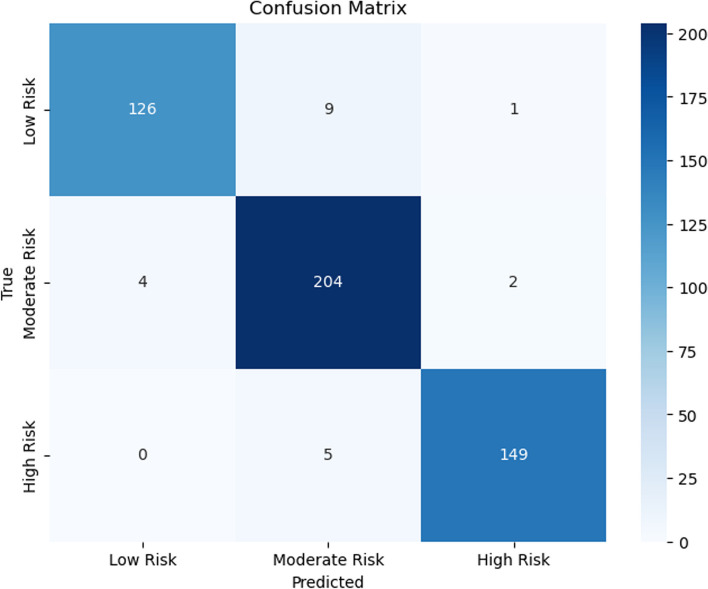
Confusion matrix plot for the muti-layer perceptron machine learning model for predicting risk groups based on DMFT

**Fig. 13:**
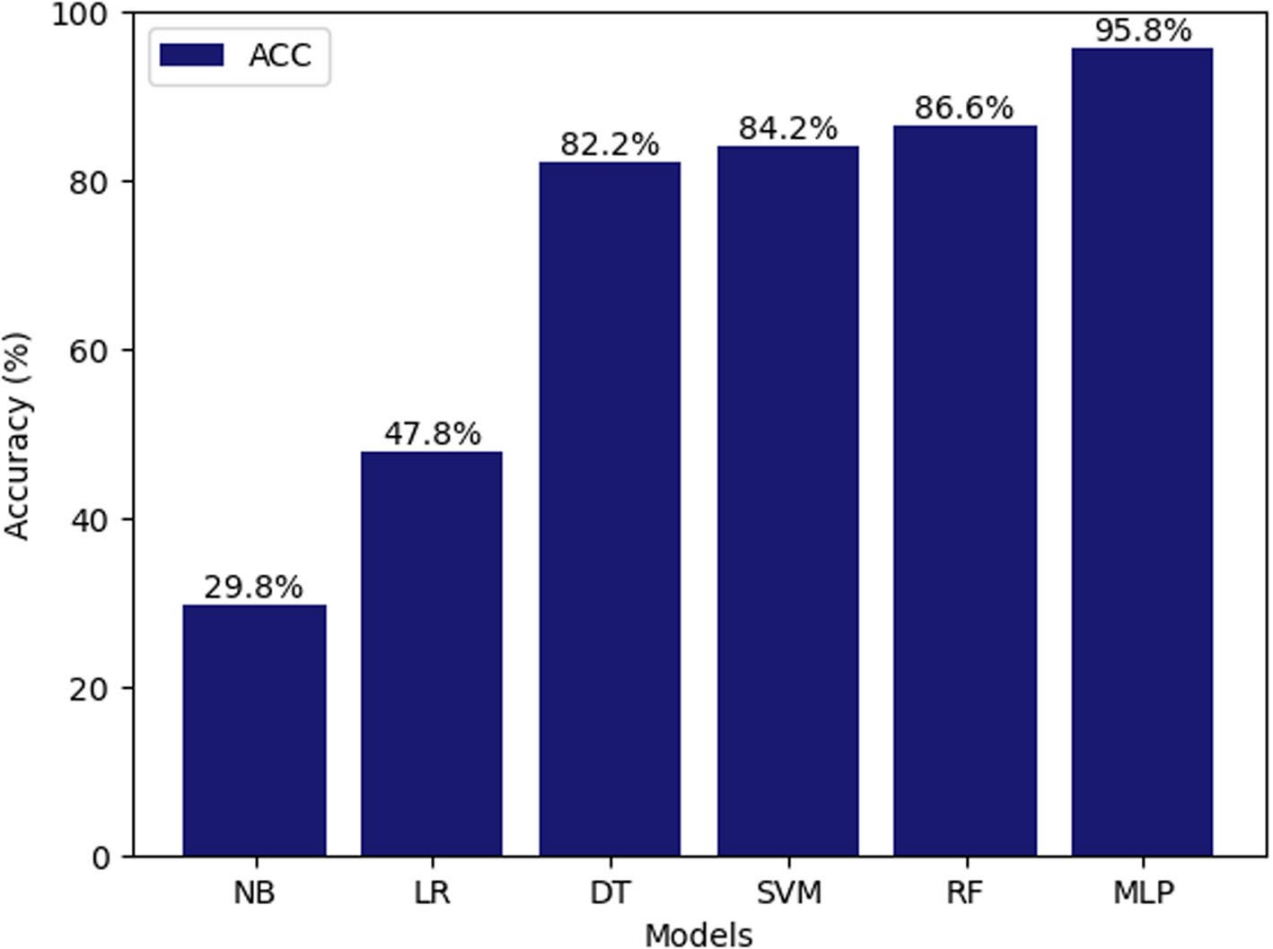
Plots of accuracy values for the prediction of risk groups based on DMFT using the naive bayes, logistic regression, support vector machine, decision tree and random forest machine learning models

Figures 7, 8, 9, 10, 11 and 12 show the confusion matrix plots of various ML algorithms including NB, LR, SVM, DT, RF and MLP used in risk group assessments for DMFT risk group prediction. The MLP model had the highest accuracy of 95.8%, while the NB model had the lowest accuracy of 29.8%, respectively (Fig. 13). The MLP model showed 96% F1-score, precision, and recall. RF model achieved 87% F1-score, precision and recall, DT model achieved 82% F1-score, precision, and recall, SVM model achieved 84% F1-score, 87% precision and 84% recall. The LR model has an F1-score of 46%, precision of 48% and recall of 48%, while the NB model has an F1-score of 19%, precision of 46% and recall of 30%. The results of the models are presented in Table 4.

**Table 4:** Precision, recall and F1 scores of naive bayes, logistic regression, support vector machine, decision tree, random forest, and muti-layer perceptron machine learning models

Models	Class	Precision (%)	Recall (%)	F1-Score (%)
Naive Bayes	Low Risk	28	94	43
	Moderate Risk	47	4	7
	High Risk	62	8	15
Logistic Regression	Low Risk	44	26	32
	Moderate Risk	47	64	54
	High Risk	53	45	49
Support Vector Machine	Low Risk	94	71	81
	Moderate Risk	74	96	84
	High Risk	98	79	87
Decision Tree	Low Risk	78	77	77
	Moderate Risk	83	84	83
	High Risk	85	84	85
Random Forest	Low Risk	88	79	83
	Moderate Risk	87	90	88
	High Risk	85	90	87
Muti-Layer Perceptron	Low Risk	97	93	95
	Moderate Risk	94	97	95
	High Risk	98	97	97

**Fig. 14:**
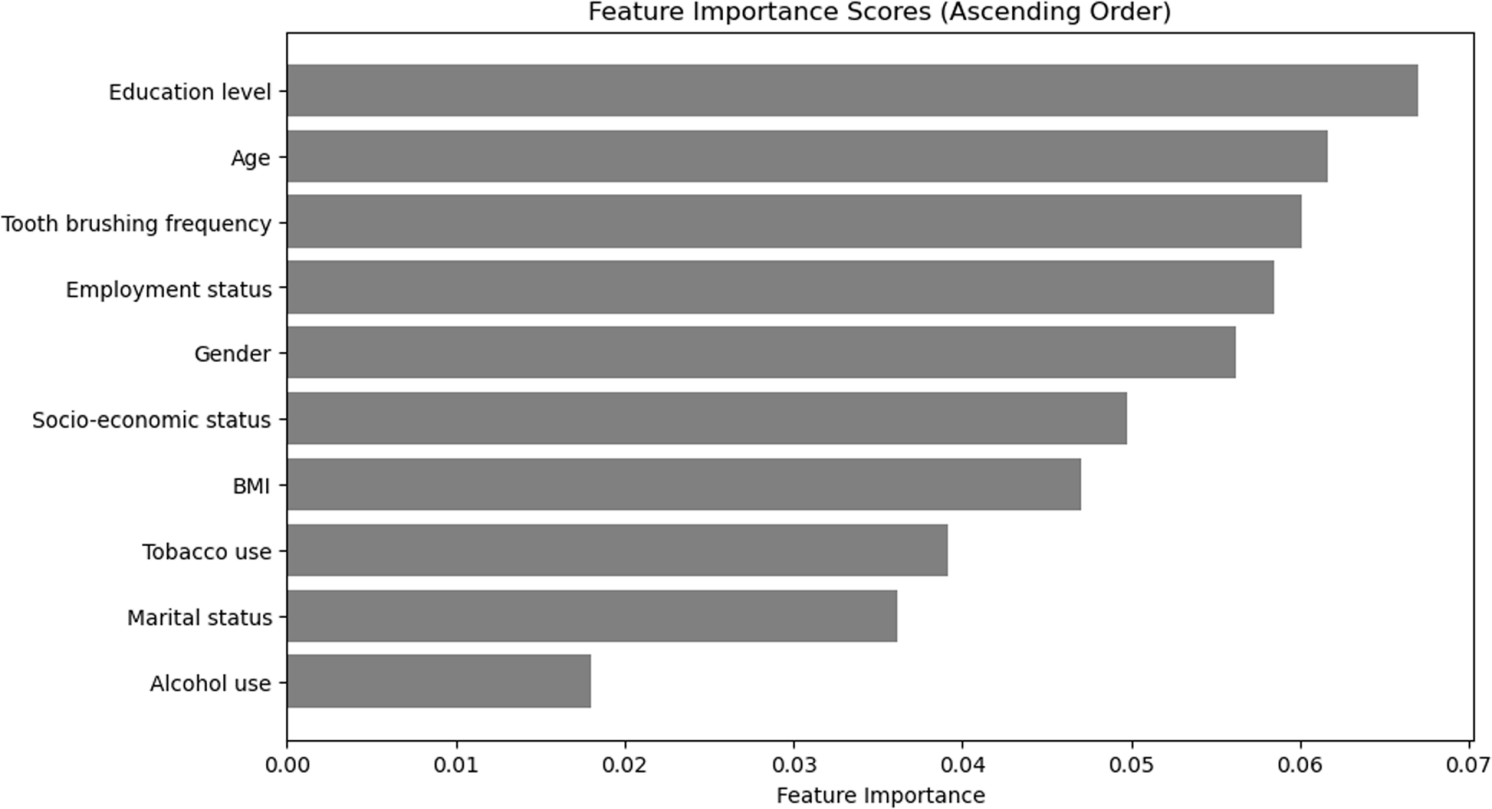
Feature importance for predicting risk groups based on DMFT, analysis of the 10 most significant features

In the importance analysis conducted to determine the most important features in the model, education level was the feature that had the greatest effect on the prediction of the model (0.061). Age was the second most important feature with a significance level of 0.056. Experiencing difficulties in performing daily life activities and walking disorder had the lowest effect. Figure 14 depicts the 10 features with the highest level of importance.These results reflect the importance analysis of the RF model. Although the accuracy rates of the other models were above 80%, the rankings remained relatively consistent.

## Discussion

The mouth serves as the entry point for the digestive system, where food is prepared for digestion with the help of the teeth. Oral health is a significant public health concern beyond personal health problems, as disorders in oral health have been linked to various diseases, including gastrointestinal system conditions [53], cardiovascular diseases [54], and diabetes mellitus [55]. Additionally, these disorders can impose a significant financial burden on countries [56]. Therefore, the assessment of oral hexalth-related risk factors is critical to the maintenance of both general and oral health.

Our study revealed that several factors contribute to a high risk of caries, including age, sex, body mass index, tooth brushing frequency, socioeconomic status, employment status, education level, marital status, hypertension, diabetes, renal disease, consumption of sugary snacks, dry mouth, and time spent in front of the TV, telephone and computer. Age is considered to be a risk factor for poor oral health, as the impact of factors causing caries and periodontal disease on teeth increases with age [57]. Studies using artificial intelligence have reported an increase in tooth loss [58], root caries [35], and early childhood caries [30, 32] with age. The findings of our study are consistent with the literature in this regard.

When dental caries incidence rates are analysed according to sex, it is generally observed that the prevalence of dental caries is greater in females than in males. This is often attributed to one of three factors: earlier tooth eruption in females, resulting in longer exposure to the caries-forming oral environment; easier access to food sources for women; and hormonal fluctuations during processes such as menstruation and pregnancy [59]. Hung et al. [35] demonstrated a significant relationship between sex and caries risk, which is similar to the findings of our study, whereas Park et al. [30] concluded that there was no significant relationship between sex. However, it is important to note that their study investigated only early childhood caries in children aged 1 to 5 years and did not consider the factors that contribute to a higher incidence of caries in women. However, it is important to note that their study investigated only early childhood caries in children aged 1 to 5 years and did not consider the factors that contribute to a higher incidence of caries in women. We believe that these differences in findings are due to the age group studied and the exclusion of relevant factors.

Overweight and obesity are major global public health problems that are characterized by excess body fat relative to lean body mass [60]. Factors strongly correlated with the predisposition to overweight and obesity include decreased physical activity, increased sedentary lifestyle, and poor dietary habits [61]. In our study, a significant relationship was found between body mass index and caries risk group. The greater and more frequent consumption of foods rich in fat and carbohydrates in overweight individuals may explain this relationship.

Among the habits affecting oral health, tooth brushing frequency and consumption of sugary snacks were found to be among the factors associated with increased risk of caries according to our study. It is important to note that the consumption of sugary snacks is a significant risk factor for caries formation, with studies showing that it increases the risk of caries fivefold [62]. This finding is supported by the findings of other studies in the literature [29, 30, 32, 58]. Flossing, which is a crucial aspect of oral health, is known to prevent root caries [63]. Although flossing was not identified as a significant caries risk factor in our study, Hung et al. [35] reported that nonusers had a greater incidence of root caries than flossers. It is important to note that their study focused only on root caries, and the limited number of flossers in our study may have contributed to the difference in findings.

Regular dental care by a professional increases the chances of early detection, prevention and treatment of oral diseases [64, 65]. Previous studies have shown that people who do not receive regular dental care from a professional have worse oral health than those who do receive regular dental care [66]. Our study revealed significant correlations between socioeconomic status, employment status, education level, and caries risk groups, which is consistent with the findings of other studies in the literature [28–30, 35, 58]. Social factors are likely to affect access to dental care, and it can be concluded that they affect oral health and caries risk.

Chronic diseases are defined as medical conditions that require a life-long course of treatment and last for more than 3 months. These diseases affect elderly people more frequently, with 80% having one chronic disease and 50% having at least two [67]. There is a relationship between oral diseases and systemic chronic diseases, with inflammation being a key factor linking most of these conditions [68]. The study revealed that caries risk groups were associated with hypertension, diabetes and chronic kidney disease. Diabetes causes periodontal damage [69] and dry mouth [70] by directly affecting the salivary glands, which has a negative impact on oral health. Hypertension indirectly exacerbates caries, as antihypertensive drugs can cause xerostomia by decreasing saliva secretion [71]. Our study found that dry mouth is a significant factor for increasing the risk of caries (*p* = 0.005). Other studies by Hung et al. [35] and Elani et al. [58] also identified diabetes mellitus and hypertension as risk factors for caries. However, unlike our study, Hung et al. [35] reported that stroke, heart disease, COPD, vision, walking and memory problems, and Elani et al. [58] reported that heart disease was also associated with an increased risk of caries. Individuals with physical and mental disabilities, such as visual impairment, inability to walk, and memory problems, may have difficulty maintaining oral hygiene. As a result, they are at an increased risk of developing caries. In our study, we found that systemic factors such as stroke, heart disease, COPD, and physical and mental disabilities, such as visual, walking, and memory problems, were not associated with caries risk groups. This may be due to the lower number of individuals with these diseases and disabilities in our study, unlike those with diabetes mellitus and hypertension.

There is a suggested correlation between spending more than 3 h in front of a screen, being married, and oral health [26, 35]. Although marital status did not directly affect caries risk, it was strongly correlated with age, which is one of the factors directly affecting caries risk (*p* < 0.00001). Additionally, married individuals may neglect personal care and oral hygiene due to their busy schedules. Increased screen time may lead to a sedentary lifestyle and unhealthy living conditions. Thus, it can be assumed that social factors such as these can indirectly increase the risk of caries.

Research indicates that the use of tobacco and the consumption of alcohol increase the risk of dental caries [72, 73]. By altering the temperature and humidity of the oral environment, smoking negatively affects the buffering capacity of saliva [74]. This altered environment causes the bacterial flora to deteriorate, leading to an increase in cariogenic bacteria [75, 76]. Similarly, toxic substances such as nicotine found in cigarettes can cause periodontal disease by affecting the immune response in the surrounding tissues [74]. Our study showed that smoking is not a contributing factor to caries risk. In the present study, we aimed to investigate the prominent indicators of dental caries at the level of the community. Therefore, we included factors that may be directly associated with dental caries, as well as other variables that may affect these factors. This study did not aim to investigate the effect of any factor alone on the risk group but rather to evaluate all factors together and select the appropriate machine learning algorithms to determine the risk group. Therefore, although tobacco use is expected to have an effect on dental caries incidence, the lack of significant results may be due to the fact that dietary habits, oral hygiene knowledge, lifestyles and social factors other than smoking vary from person to person. Additionally, alcohol consumption may increase host susceptibility to infections such as periodontitis because ethyl alcohol increases susceptibility to infections by impairing the function of neutrophils, macrophages and T cells [77]. In addition to its direct effects, poor oral hygiene in alcoholic patients is one of the main effects of alcohol on oral health [78]. In our study, alcohol consumption was not found to be a significant risk factor for caries. This is probably due to its low prevalence (7.7%) in our study group. In contrast to our findings, Hung et al. [35] reported that tobacco and alcohol use significantly contribute to the risk of root caries. Most of the machine learning studies in the literature dealt with caries risk in children. Smoking status and alcohol consumption were not evaluated. For this reason, as there are no studies in the literature that have evaluated this variable in adults, our results could not be discussed with another study other than the study by Hung et al. [35]. We recommend that future studies should evaluate the effect of smoking on the risk of caries in adults.

Machine learning is being used in oral health to provide dentists with a tool to improve the oral health status of individuals, enabling them to make early decisions to prevent dental caries and thus improve overall quality of life. There are many studies in the current literature using machine learning techniques to assess oral and dental health. Kang et al. [26] collected data from a child oral health survey conducted by the Korean Centre for Disease Control and Prevention in 2018 and created a dental caries prediction model using the RF, gradient boosting decision tree (GBDT), SVM, LR, artificial neural network, convolution neural network, and long short-term memory machine learning algorithms. RF achieved the highest performance compared to the other machine learning methods, with 92% accuracy, 90% F1-score, 94% precision and 87% recall. As in this study, the RF algorithm was very successful in our study with 86% accuracy, 87% F1-score, 87% precision and 87% recall.

Kang et al. [27] conducted another study with the same dataset and used GBDT, RF, LR, SVM and long short-term memory algorithms; GBDT achieved the highest success, with an accuracy, F1-score, precision and recall of 95%, 93%, 99% and 88%, respectively. In this study, the DT model achieved 82% accuracy, 82% F1-score, 82% precision and 82% recall.

Ramos-Gomez et al. [28] analysed the answers given by the parents or caregivers of children to questions asked to predict the probability of dental caries in children aged 2–7 years using the RF machine learning algorithm and obtained accuracy rates of 62% and 73% for active caries and caries history, respectively.

Sadegh-Zadeh et al. [29] sampled a total of 780 parents and children under the age of five to assess the risk of dental caries in children aged 5 years and under. They employed ten different machine learning modeling techniques to build a highly accurate classification model to predict caries risk with this data and showed that RF and MLP machine learning models had the best accuracy of 97.4%. In our study, as in this study, the MLP model was the most successful model with 96% accuracy.

Hung et al. [35] used data from the 2015–2016 National Health and Nutrition Examination Survey to predict root caries and revealed that the SVM algorithm performed best, with 97% accuracy, 94% specificity, 95% precision and 99% recall, for identifying root caries. In our study, this algorithm demonstrated 84.2% accuracy, 84% F1-score, 84% precision and 46% recall.

Park et al. [30] analysed the data of 4195 children between 1 and 5 years of age from the Korean National Health and Nutrition Examination Survey (NHANES) data from 2007 to 2018 for the prediction of early childhood caries using the LR, XGBoost, RF and LightGBM algorithms and calculated the model with the highest accuracy rate among the four prediction models as the LR with an accuracy rate of 76%. The LR model achieved 47% accuracy in the present study.

Yang et al. [31] used linear regression and RF classifier machine learning algorithms to estimate the DMFT scores of 12-year-old children and reported prediction accuracies of 15.24% and 43.27%, respectively.

Kumar et al. [79] utilized machine learning algorithms, including RF, DT, LR and NB, to provide a model for dental caries detection and showed that DT provided a more accurate model with an accuracy level of 85.62%. The NB model, which was also used in this study, showed 77% accuracy, 85% F1-score, 80% precision and 90% recall in this study and 29% accuracy, 19% F1-score, 46% precision and 30% recall in our study, making it the least successful model in both studies.

Qu et al. [32] used the LR, RF and AdaBoost algorithms to create an early childhood caries risk prediction model based on behavioural factors and showed that the RF model had the highest accuracy (82%).

Elani et al. [58] conducted a study using extreme gradient boosting trees, RF, neural networks, a light gradient boosting machine, and LR models to determine the socioeconomic predictors of tooth loss and reported that the RF model achieved the highest performance, with an accuracy rate of 84.3% for edentulism.

Karhade et al. [33] used Google Cloud AutoML to develop an automated machine learning algorithm to classify children according to early childhood caries status, and the model considering only 2 variables (child's oral health status and child age) showed a high accuracy rate of 67%.

Wang et al. [34] used machine learning algorithms, including the extreme gradient boosting and NB algorithms, to predict the oral health status index score and referrals for treatment needs (RFTN) in children aged 2–17 years. They used random bootstrap samples with manually added Gaussian noise and achieved 93% recall and 49% specificity in predicting RFTN.

Kang et al. [26], Sadegh-Zadeh et al. [29], Elani et al. [58], Yang et al. [31] and Qu et al. [32] found that RF is the machine learning model with the highest success rate. In our study, this model was the second most successful model after the MLP algorithm. Additionally, we found that the DT machine learning model has an accuracy of over 80%, which is also consistent with the findings of Kang et al. [26] and Kumar et al. [79].

Some of our features exhibited a low correlation level with DMFT, prompting us to explore feature selection. During the feature selection process, we considered the correlation of independent variables with DMFT. Initially, we set the correlation threshold at 0.1. In this case, variables such as age, tooth brushing frequency, socio-economic status, employment status, education level, and marital status remained in the dataset, while other independent variables were excluded. With a correlation threshold of 0.1, the accuracy rates for NB, LR, SVM, DT, RF, and MLP models were 52%, 48%, 44%, 53%, 54%, and 52%, respectively.

Subsequently, we adjusted the correlation threshold to 0.05. This time, 18 independent variables, including age, gender, BMI, tooth brushing frequency, socio-economic status, employment status, education level, marital status, hypertension, diabetes, renal failure, consumption of sugary snacks, frequency of snack consumption, radiotherapy history, dry mouth, time spent in front of the TV, phone, or computer, and walking disorder were included, while 12 independent variables with a correlation with DMFT lower than 0.05 were excluded. In this case, the accuracy rates for NB, LR, SVM, DT, RF, and MLP models were 38%, 48%, 45%, 74%, 76%, and 77%, respectively.

In both scenarios, there was a slight increase in accuracy for NB and LR models. However, notably, when the correlation threshold was set at 0.1, SVM, DT, RF, and MLP models exhibited dramatic decreases in accuracy. As a result, the highest level of accuracy was achieved when all independent variables were included without feature selection.

This study is distinctive from other studies in the literature because it focuses on caries risk group assessment rather than caries presence. This approach goes beyond existing studies and offers a more effective strategy for identifying the caries potential of individuals and taking preventive measures in advance. In addition, to the best of our knowledge, this is the first study to address oral health risk groups in adults with machine learning algorithms. Another valuable advantage of this study is that it clearly demonstrates the relationships between oral health risk factors of individuals and the interactions of these factors with DMFT risk groups. However, interpreting these relationships only from the table may lead to misleading results, as all the other variables are not equal. Furthermore, this study reflects only the dietary and social practices of the Turkish population.

The prospective collection of the data used in our study can be considered one of the limitations of the study because of the limited dataset. From a scientific point of view, using larger datasets may increase the strength of the general validity of the findings obtained in the study.

## Conclusion

Caries risk assessment using a simple questionnaire makes it possible to identify individuals at risk of caries, to determine the most significant risk factors and to provide follow-up information that can help to reduce the risk of caries over time. It is also extremely important for the application of effective preventive treatments and for the prevention of general health problems caused by the deterioration of oral health. Recently, artificial intelligence has become a popular tool for evaluating the risk of caries. In this study, we used machine learning algorithms to determine the caries risk group in adults. The MLP and RF algorithms showed high accuracy in determining the caries risk group. This study highlights the potential of machine learning algorithms in this area, which is promising for future research.

## Data Availability

The datasets used and/or analysed during the current study are available from the corresponding author upon reasonable request.

## Abbreviations

DMFT: Decayed, Missing and Filled Teeth
NB: Naive Bayes
LR: Logistic Regression
SVM: Support Vector Machine
DT: Decision Tree
RF: Random Forest
MLP: Multi-Layer Perceptron
ANN: Artificial Neural Network
COPD: Chronic obstructive pulmonary disease
ADHD: Attention deficit hyperactivity disorder
GBDT: Gradient Boosting Decision Tree
RFTN: Referral for Treatment Needs
